# Eating Disorders in Boys and Men: A Gender‐Critical Narrative Review on Recognition, Stigma, and Treatment

**DOI:** 10.1002/eat.70085

**Published:** 2026-03-24

**Authors:** Georgios Paslakis

**Affiliations:** ^1^ University Clinic for Psychosomatic Medicine and Psychotherapy, Medical Faculty, Campus East‐Westphalia, Ruhr‐University Bochum Bochum Germany

**Keywords:** body dissatisfaction, boys, eating disorders, epidemiology, gender, help‐seeking, intersectionality, men, psychotherapy

## Abstract

In this narrative review, I highlight diversity‐related developments in eating disorder (ED) research, situate my own program of work on adult men within the broader literature on boys and men, and focus on risk factors, symptoms, diagnosis, and treatment in boys and men. I build on recent reviews on gender in EDs but adopt a gender‐critical perspective that asks why males remain comparatively invisible in epidemiology, diagnosis, and services despite their substantial burden of illness. EDs in males span the full diagnostic spectrum and often present with muscularity‐oriented body dissatisfaction, compulsive exercise, and performance‐enhancing substance use that are poorly captured by traditional, thinness‐focused tools. Internalized stigma regarding the construction of EDs as “women's disorders” and traditional masculinity norms create gender‐specific barriers to recognition and help‐seeking in males. Minority stress (i.e., stress related to discrimination, stigma, or social rejection among sexual and gender minorities) can further shape the manifestation and maintenance of EDs in sexual and gender minority males. Given the emerging evidence for distinct male phenotypes and pathways into care, there is a high need for gender‐sensitive diagnostics, treatment settings, and health policies that make EDs in males more visible, more intelligible, and more treatable across developmental stages.

## Summary

- Boys and men are underrepresented in eating disorder research and care.
- Internalized stigma and masculinity norms may impair recognition and help‐seeking in males with eating disorders.
- Muscularity concerns appear to be key drivers for eating disorders in boys and men.
- Treatment and policy need to represent diverse masculinities and explicitly target gendered barriers to care.

## 1 Introduction

In this narrative review, I adopt a deliberately gender‐critical perspective on eating disorders (EDs) in boys and men that is shaped by my own empirical work and clinical experience. Rather than providing an exhaustive overview of the literature, I build on existing reviews of gender differences in EDs (e.g., Capuano et al. 2025) to ask why males remain comparatively invisible in epidemiology, diagnosis, and treatment, despite their substantial burden of illness. I focus on how masculine role expectations, gendered stigma, and societal body ideals intersect with developmental factors from adolescence onwards to shape recognition, clinical presentation, help‐seeking, and treatment outcomes in males with EDs.

## 2 Epidemiology

EDs are a growing public health problem and one of the leading mental disorders to cause disability adjusted life years (GBD 2019 Mental Disorders Collaborators. 2022). Several reviews specifically examined EDs in boys and men (“males”) and convincingly demonstrated that males are affected across the diagnostic spectrum but remain underrecognized, underrepresented in research, and underserved in treatment (Brown and Keel 2023; Burnette et al. 2022; Capuano et al. 2025; Nagata et al. 2020). Epidemiological data show that individuals of all genders, sexual orientations, ages, ethnicities, and socioeconomic backgrounds may be affected by EDs (Halbeisen, Brandt, et al. 2022). Also, even though the absolute burden of EDs is higher in girls and women (“females”) than in males, the relative increase in males over the last decades appears to be more pronounced compared to females (GBD 2019 Mental Disorders Collaborators. 2022), and population‐based data show that across all ED diagnoses up to one in four clinical cases occurs in boys or men (Galmiche et al. 2019; GBD 2019 Mental Disorders Collaborators. 2022). The evidence underscores that EDs in males emerge early in the life course and supports the need for gender‐sensitive detection and intervention efforts from adolescence onwards (Brown and Keel 2023; Capuano et al. 2025; Nagata et al. 2020).

The prevalence of EDs has been repeatedly shown to be higher in gay or bisexual males compared to heterosexual men and higher in trans men than in trans women and cis women (Nagata et al. 2020; Rasmussen et al. 2023). There are several theoretical models to explain these differences in prevalence within underrepresented groups. Chronic exposure to minority stressors such as discrimination and rejection fosters shame and negative affect; the internalization of dominant appearance norms may amplify discrepancies between one's actual body and both ideal and ought standards dictated by mainstream and in‐group aesthetics, and pervasive racialized and sexual objectification and gendered body surveillance promote an external observer's gaze on the body, further increasing body shame and self‐objectification (Rodgers et al. 2025). For example, disordered eating may be a strategy to reduce painful self‐discrepancies and to cope with or preempt stigmatization and dehumanization in sociocultural contexts that narrowly define beauty, gender conformity, and desirability (Hendricks and Testa 2012). Intra‐minority stress has also been identified as a risk factor for the manifestation of EDs (Soulliard et al. 2025). Although prevalence estimates are higher in sexual and gender minority males, the absolute majority of males with EDs in the general population are cisgender and heterosexual, reflecting the underlying population distribution of sexual and gender identities.

Taken together, the epidemiology of EDs in males is less a story of rarity than of selective visibility: boys and men are present across diagnostic categories and muscularity‐oriented presentations but are rendered less visible by gendered assumptions about who “should” have an ED. When disorders are culturally coded as feminine, male cases drop out of surveillance, clinical pathways, and even self‐recognition, which means that epidemiological figures partly reflect what systems are prepared to see, rather than the true distribution of pathology (Halbeisen, Brandt, et al. 2022).

## 3 Clinical Presentation

The differences in the clinical presentation of EDs between males and females may be seen in connection with gender‐distinct sociocultural perceptions of the body (Bartel et al. 2026). While girls or women mostly focus on the pursuit of thinness, boys or men are more oriented toward muscularity ideals (Halbeisen et al. 2024). In one of the most comprehensive network analyses to date, we found that the pursuit of muscularity was central to the identification of EDs in men (Eschrich et al. 2025). Yet, the pursuit of masculinity is not included in current diagnostic tools for EDs, which were developed and validated for females. In the first factor‐analytic validation of the Eating Disorder Examination‐Questionnaire (EDE‐Q) in men diagnosed with a clinical ED, we found that concerns related to weight gain and body dissatisfaction may need to be considered as separate dimensions in men, with body dissatisfaction possibly being attributable to concerns related to muscle mass (Laskowski et al. 2023). Thus, the body mass index (BMI) appears to be of limited significance as a diagnostic criterion for men; eating pathology is easily overlooked when weight alone appears “normal” or even elevated in muscle‐oriented men.

Muscular body ideals act as an expression of perfectionist, performance‐related norms that are reinforced by fitness culture and social media with so‐called “fitspiration” content (Patarinski et al. 2025; Vintró‐Alcaraz et al. 2025). Males with a strong focus on muscularity and high sensitivity to social evaluation are particularly at risk: sociocultural influences increase body dissatisfaction, which, in turn, may promote the development of restrictive or other dysfunctional eating behaviors (Ganson et al. 2025; Halbeisen et al. 2024; Laskowski et al. 2025; Zaiser et al. 2024). Internalized masculinity norms that emphasize control, strength, and independence can also inhibit adaptive emotion regulation and promote ED‐related behavioral patterns (Lehe et al. 2024). Boys and men, compared to girls and women, are more likely to engage in excessive weight training and rigid dieting patterns based on high protein intake to increase muscle volume, followed by periods of food restriction or even fasting and/or binging–purging to reduce body fat, including the use of dietary supplements and performance‐enhancing substances like androgenic anabolic steroids (AAS) (Laskowski et al. 2025; Patarinski et al. 2025). In a systematic review, we showed that males use AAS more frequently than females, and that disturbed body image is a reliable predictor of AAS use (Zaiser et al. 2024). Although such muscle‐enhancing behaviors and appearance outcomes are considered healthy or an expression of discipline and perseverance and are socially positively reinforced—making it hard to recognize an underlying ED—they can take on pathological traits and lead to lasting negative consequences for the mind, body, and well‐being. Men who use drugs to build muscle or lose weight have both a higher rate of EDs and a higher risk of medical complications, hospitalizations, and death (Or et al. 2019). Among males, gay boys or men show a higher rate of body dissatisfaction and dysfunctional behaviors to achieve the so‐called mesomorphic body ideal (Zaiser et al. 2024). Accordingly, muscle dysmorphia (MD), characterized by the belief that one is not muscular enough, even in those with “normal” muscle development or who are objectively muscular, is often comorbid with EDs in males (Vasiliu 2023). In a survey conducted in Germany, we found rates for probable MD in men from the general population to be approximately 8 times higher than those in women (Zaiser et al. 2026).

From a sociocultural perspective, these body projects of control, optimization, and self‐discipline are not simply individual vulnerabilities but highly gendered responses to broader social demands. Contemporary growth‐society ideals reward males who treat their bodies as perpetual improvement projects—stronger, leaner, more efficient—while pathologizing visible vulnerability, softness, or dependence. For many boys and men, restrictive eating, compulsive exercise, and AAS use become culturally sanctioned ways of performing masculinity under conditions of pervasive performance pressure and insecurity, even as these same practices slide into clinically significant eating pathology (Bartel et al. 2026; Mokhwelepa and Sumbane 2025).

## 4 Help‐Seeking

If epidemiological data show where males with EDs exist, help‐seeking patterns show where they disappear. Men are often ambivalent about revealing their ED (Richardson and Paslakis 2021). Social comparison processes, (self‐)stigmatization, shame, and emotional suppression act as transdiagnostic factors that can exacerbate EDs and depressive and compulsive symptoms and make it difficult to seek help (Lehe, Halbeisen, Juergensen, et al. 2025; Patarinski et al. 2025). The perception of EDs as “women's disorders” or disorders mostly affecting gay men conflicts with traditional ideals of masculinity, and openly dealing with an ED can be accompanied by the fear of losing one's standing as a man (Lehe et al. 2024; Lehe, Halbeisen, Juergensen, et al. 2025). In fact, this stigma, internalized, may prevent help‐seeking. In one of our studies, we found that the more severe the ED symptoms were, the more willing men were to seek help, an association that was, however, moderated by the internalized stigma of EDs as a women's disease: the greater the internalized stigma, the less likely men were to seek help in response to ED symptoms (Lehe et al. 2024). In another study with a similar design, in which we included both men and women, we found this result to be gender‐specific, that is, to only apply to men (Lehe, Halbeisen, Juergensen, et al. 2025). This suggests that gendered stigma is a specifically male mechanism of invisibilization: precisely those men with more severe ED symptoms may be least likely to seek help when they experience their eating problems as incompatible with being a man.

The misconception of EDs as women's disorders appears to be widespread among healthcare professionals as well. It is therefore important that physicians of all specialties, and healthcare practitioners with primary care responsibilities in particular, acquire basic competencies for tackling EDs in males appropriately. In a project aimed at destigmatizing EDs in men, we built six 5‐min videos on various aspects of EDs in men, addressing general practitioners (GPs) in Germany (Lehe, Halbeisen, and Paslakis 2025). We sought to sensitize primary care physicians to the occurrence and manifestation of EDs in men, to impart knowledge and specific skills, and thus to combat the stigmatization of EDs in men. In a randomized design with an intervention and waiting list control group, we were able to show that our knowledge‐based intervention contributed to a significant increase in both GPs' knowledge and self‐efficacy in dealing with men with EDs, which we attributed to the reduction of cognitive stigma (i.e., increase in knowledge) and reduction of behavioral stigma (i.e., increase in competency to deal with the disorder) (Lehe et al. 2026).

## 5 Treatment and Outcomes

Treatment services and information materials for EDs are still mostly directed at girls or women (Richardson and Paslakis 2021). Being the only male in a treatment setting may lead to inner withdrawal in group therapies and facilitate experiences of exclusion. Yet, the current evidence does not allow conclusions on what treatment environments for males could or should look like (Brown and Keel 2023). Existing treatments and the settings in which they are delivered could become more effective and acceptable to males if they could be adapted to reflect and support their specific motivations and needs. Males' socialization interferes with psychotherapy (Solmi et al. 2021). Male norms emphasize independence, and boys or men may have little experience with and/or feel uncomfortable expressing their feelings or examining their symptoms in depth. Feeling exposed or even vulnerable and needy can violate the code of masculinity. It is therefore important that professionals look for ways to increase males' willingness to undergo treatment as needed.

Clinical studies show that men, when their access to treatment is comparable to women's and adequate diagnostics are applied, may achieve similar symptom amelioration and remission rates to women—in some studies even more favorable, even though they often start therapy with a delay (Halbeisen et al. 2024), while calling for more robust gender‐disaggregated analyses. The evidence on gender‐specific treatment outcomes is primarily based on studies on BED with mixed‐gender samples and must be considered significantly limited and preliminary. Only one randomized clinical trial (RCT) has thus far included mixed‐gender individuals with BN (Hildebrandt et al. 2017) and AN (Cardi et al. 2024) with *n* ≥ 20 men. In a retrospective design, we used admission, follow‐up, and discharge data from over 2000 patients to systematically compare samples of men and women with different ED diagnoses who were similar in terms of diagnosis, age, and treatment duration. Under these conditions, men, especially those with AN and BED, benefited more from treatment than women. Men with AN gained weight significantly more pronouncedly and faster than their female counterparts and also showed greater improvement in ED‐associated psychopathology. Men with BED lost significantly more weight than women—despite the fact that weight loss did not constitute an explicit goal of treatment (Halbeisen, Braks, et al. 2022). The reasons for such differences in outcomes remain unclear, warranting further study. For example, they could be due to sex‐related (physiological) differences, gender‐associated coping styles, personality traits, treatment adherence, or other treatment‐related characteristics. At the same time, recognizing that most existing interventions, trial cohorts, and treatment settings were designed with women in mind lets us think that men who do enter treatment may have already overcome substantial structural barriers. Through this lens, findings that men do as well as or better than women in treatment may reveal as much about who manages to reach treatment as about intrinsic gender differences in treatment response and underscore the need to redesign trials and services so that help‐seeking becomes an accessible option for a much broader range of boys and men.

As it is difficult to recruit a sufficient number of boys or men to conduct prospective treatment outcome studies, we are currently conducting an individual patient data (IPD) meta‐analysis using pooled global data. Several research groups around the world have agreed to provide us with data from their RCTs that also included men (Halbeisen et al. 2025). Researchers who are interested in becoming part of our “MAN‐UP” consortium and are willing to share RCT data or effect sizes from studies that included men are very welcome and kindly asked to reach out to our research group via the author's email.

Fortunately, the importance of research to take into account the subjective (lived and living) experiences of people with EDs is increasingly becoming recognized. Current research approaches underscore the need for a change in perspective and closer collaboration in the study and treatment of EDs with those affected by them. The goal is to expand empirical research, diagnostic criteria, and quantitative analyses by incorporating contributions from affected individuals and integrating interdisciplinary approaches (Brandt et al. 2025). The narratives of shared lived and living experiences contribute meaningfully to promoting empathy, understanding, self‐reflection, and commitment to interventions for the treatment of EDs, thus having a more profound impact on individuals dealing with EDs. Remembering that males are not a homogeneous category but have a variety of identities and experiences is essential to guard against the pitfalls of stereotyping and to make future efforts as inclusive as possible of the needs of boys and men with EDs in all dimensions.

## 6 Conclusion and Outlook

Gender is one of many social variables in the field of mental health that must be taken into account in clinical practice and research, here ED research. There is much more knowledge about EDs in boys and men that we need to gain. The ideal of a muscular body is sought after by many boys and men. Future research on ED in boys and men must move beyond female‐derived models to develop gender‐sensitive diagnostics that adequately capture muscularity‐oriented body dissatisfaction and pathology not reflected by BMI alone. Our screening and diagnostic tools for all genders with EDs need to be refined and validated. Due to prevailing stigma toward men with EDs, affected men may not seek treatment; thus, taking gender‐specific aspects into account may facilitate treatment. Future research must not only address phenotypic differences or collect epidemiological data, but also identify the underlying pathomechanisms related to the conceptions of gender (e.g., the role of gender norms). It is also important to not only focus on gender‐specific group differences but also on within‐group diversity to better understand the manifestation and maintenance of EDs. This diversity includes developmental trajectories from adolescent boys to adult men, with early onset of disordered eating, muscularity concerns, and minority stress shaping later clinical presentation and treatment needs. Large, collaborative, international studies are needed to generate sufficiently powered primary data on men's treatment trajectories and outcomes, including marginalized ethnic, sexual, and gender minority groups. This requires routine inclusion of males in clinical trials, systematic collection of gender‐relevant variables, and integration of minority stress, self‐discrepancy, and objectification frameworks into study designs. Policymakers should mandate gender‐inclusive guideline development, fund men‐focused and intersectionality‐informed ED research, and ensure that screening, training, and services in primary and specialized care explicitly target men's stigma and help‐seeking barriers. Finally, public health strategies and information materials must be revised to represent men and diverse masculinities, normalize EDs across genders, and embed early detection of muscularity‐driven risk in different contexts like schools, sports, and primary care. More fundamentally, a gender‐sensitive approach to EDs in males requires us to reflect critically on the social conditions under which these disorders manifest. In cultures that idealize permanent self‐optimization, productivity, and control, many boys and men learn early to treat their bodies as projects and to keep vulnerabilities private. EDs in males are, therefore, not only misfits within a female frame but can also be understood as expressions of a social context that makes acknowledgment of need and genuine care difficult to claim.

## Lived Experience Involvement Statement

No specific efforts were undertaken to involve persons with lived experience in the study design or execution, or in the preparation of this manuscript.

## Funding

The author has nothing to report.

## Ethics Statement

The author has nothing to report.

## Consent

The author has nothing to report.

## Conflicts of Interest

The author declares no conflicts of interest.

## Data Availability

The author has nothing to report.
